# Supplementary material for: Higher resolution pooled genome-wide CRISPR knockout screening in Drosophila cells using Integration and Anti-CRISPR (IntAC)
Source: bioRxiv. 2024 Sep 25:2024.09.19.613976. Preprint. [Version 2] doi: 10.1101/2024.09.19.613976 (PMC11429967; doi:10.1101/2024.09.19.613976)
Supplement: 1 [file NIHPP2024.09.19.613976V2-supplement-1.pdf]

bioRxiv preprint doi: https://doi.org/10.1101/2024.09.19.613976; this version posted September 25, 2024. The copyright holder for this preprint (which was not certified by peer review) is the author/funder, who has granted bioRxiv a license to display the preprint in perpetuity. It is made available under aCC-BY 4.0 International license.

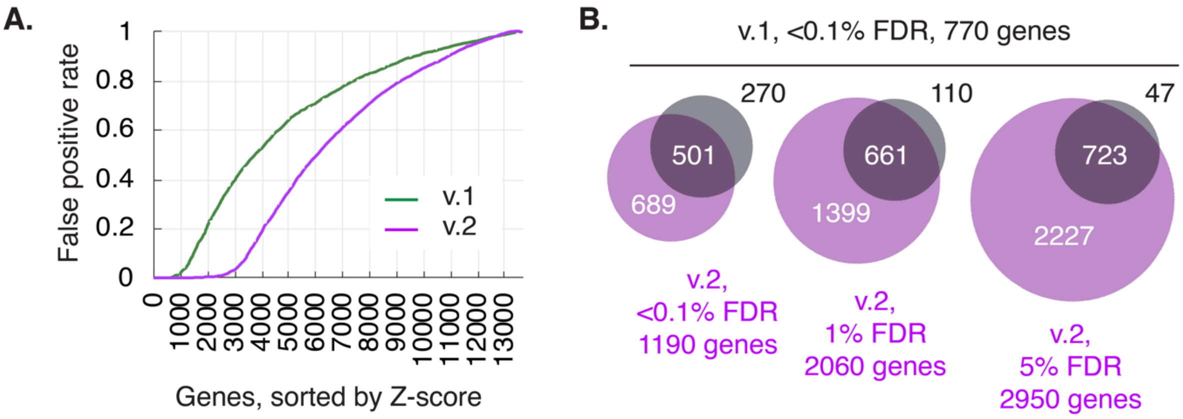


**Supplementary Figure 1: Comparison of v.1 and v.2 screens.** V.2 screens produce a larger set of fitness genes compared with v.1. (A) Cumulative distribution of false-positive rate (using non-expressed genes as false-positives, FKPM<1) as a function of genes, sorted in ascending value by gene Z-score (greater-to-less likelihood of being essential for cell fitness). (B) The 770 genes with the strongest likelihood of representing bona fide fitness genes in v.1 screens are mostly a subset of those detected in v.2. The overlap becomes progressively clearer when the false-discovery rate (FDR) of v.2 fitness genes is relaxed.

bioRxiv preprint doi: https://doi.org/10.1101/2024.09.19.613976; this version posted September 25, 2024. The copyright holder for this preprint (which was not certified by peer review) is the author/funder, who has granted bioRxiv a license to display the preprint in perpetuity. It is made available under aCC-BY 4.0 International license.

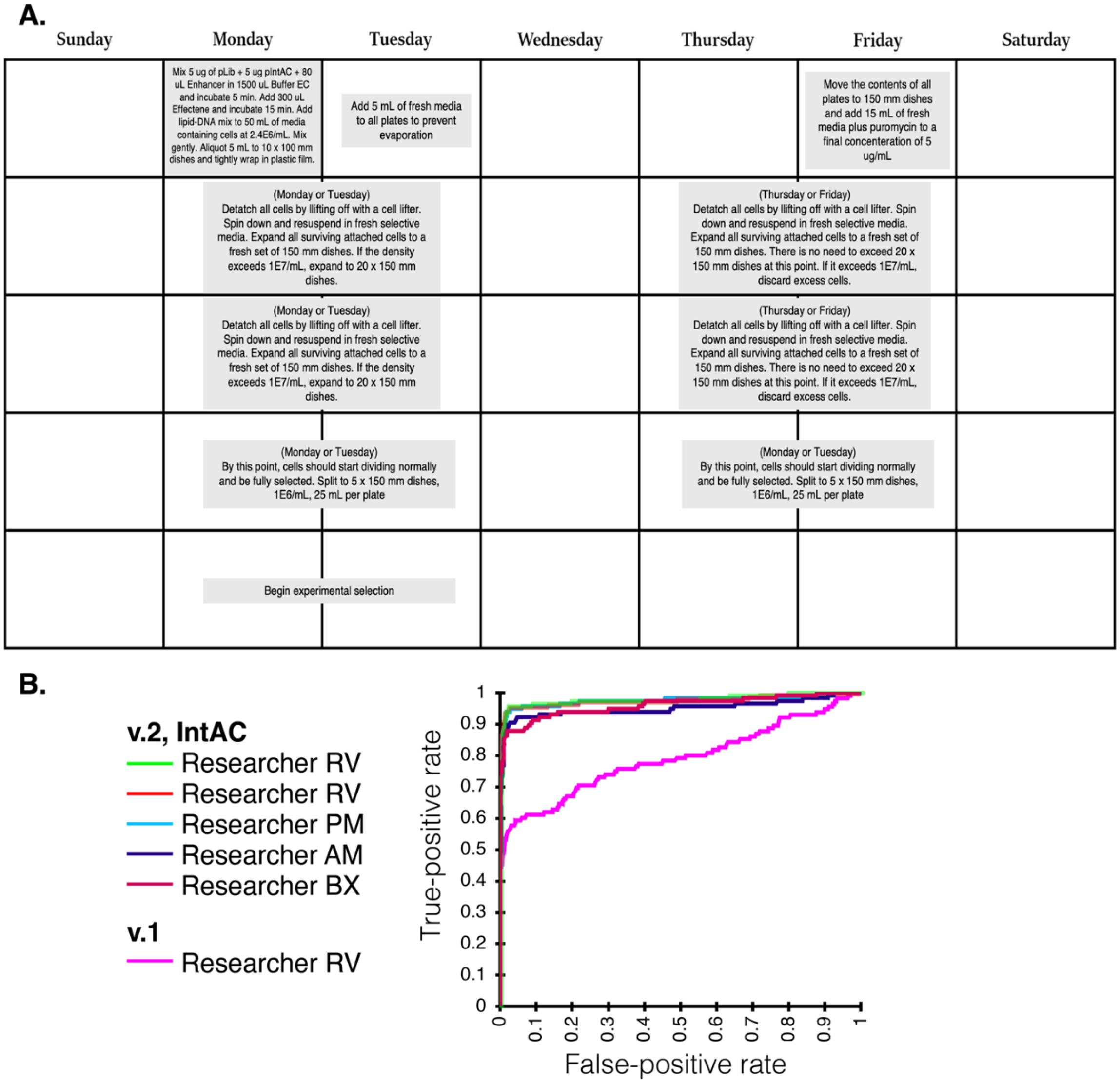


**Supplementary Figure 2: Validation of IntAC CRISPR screen across independent trials and researchers.** (A) Hands-on steps for generating a pooled library using IntAC. (B) Performance of the IntAC platform in independent trials by different researchers. Data from multiple independent screens conducted using the IntAC platform shows high reproducibility in terms of sgRNA dropout patterns and gene Z-scores. A precision-recall analysis comparing the detection of essential genes (true-positives, KEGG-assigned ribosome and proteasome genes) versus false-positives (non-expressed genes, genes with mRNA FPKM < 1). These independent trials confirmed the high precision and robustness of the v.2, IntAC, system relative to the previous v.1 CRISPR screening system in the hands of different researchers

bioRxiv preprint doi: https://doi.org/10.1101/2024.09.19.613976; this version posted September 25, 2024. The copyright holder for this preprint (which was not certified by peer review) is the author/funder, who has granted bioRxiv a license to display the preprint in perpetuity. It is made available under aCC-BY 4.0 International license.

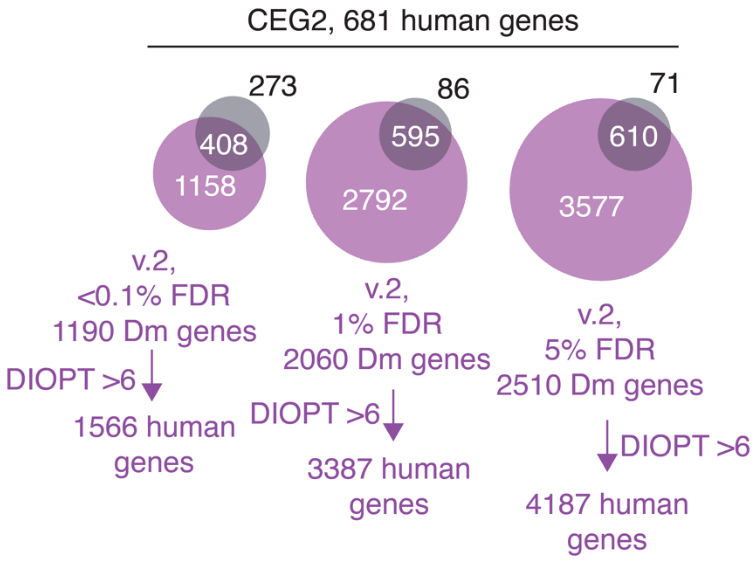


**Supplementary Figure 3: Overlap between human cell essential geneset and *Drosophila* cell essential geneset.** The 681 genes in the core essential geneset v.2 [49]. The overlap becomes progressively clearer when the false-discovery rate (FDR) of v.2 fitness genes is relaxed.

bioRxiv preprint doi: https://doi.org/10.1101/2024.09.19.613976; this version posted September 25, 2024. The copyright holder for this preprint (which was not certified by peer review) is the author/funder, who has granted bioRxiv a license to display the preprint in perpetuity. It is made available under aCC-BY 4.0 International license.

**Supplementary Table 1.** Raw and processed data for IntAC CRISPR screens. The 'sgRNA-level' tabs contain the unprocessed count data from two different screens. For the fitness screen, the count data was analyzed using the MAGeCK 'maximum likelihood estimation (MLE)' package to calculate Z-scores. These Z-scores indicate the likelihood that a given gene was enriched (positive Z-score) or depleted (negative Z-score) in the cell pools [26]. The results are presented in the 'gene-level' tab. Additionally, mRNA expression data for each gene, as compiled by modENCODE [27], is provided. For the cytidine overload screen, we used the 'test' package in MAGeCK [26] to calculate the median log2 fold-change for the 10 sgRNAs targeting each gene. This information is also included in the 'gene-level' tab.

**Supplementary Table 2. Putative fitness gene groups in human cells identified by the essentiality of their unique *Drosophila* ortholog.** We present 123 genes scoring as essential for *Drosophila* cell fitness (false-discovery rate < 5%) for which the putative human orthologs (DIOPT score > 6 [30]) do not score as essential for human cell fitness. For this analysis, we report only gene families for which the minimum first quartile CERES value is greater than -0.5, indicating that none of the human orthologs is strongly essential for cell fitness in the DepMap [31].
